# Studying latent change process in height growth of children in Ethiopia, India, Peru and Vietnam

**DOI:** 10.1186/s12887-022-03269-3

**Published:** 2022-04-14

**Authors:** Senahara Korsa Wake, Temesgen Zewotir, Essey Kebede Muluneh

**Affiliations:** 1 College of Science, Bahir Dar University, Bahir Dar, Ethiopia; 2 School of Mathematics, Statistics and Computer Science, University of KwaZulu-Natal, Durban, South Africa; 3 School of Public Health, Bahir Dar University, Bahir Dar, Ethiopia

**Keywords:** Basis coefficient, Freed-loading, Growth curve, Latent variable, Longitudinal data, Structural equation modeling

## Abstract

**Background:**

Anthropometric measurements of healthy children differ in different parts of the world due to the diverse ethnicity and cultural backgrounds of families. In longitudinal studies, appropriate modeling of repeated anthropometric measures can improve the understanding of patterns of change, determinants of patterns, and variations in patterns of change over time. The objective of this study was to examine the latent change in physical height of children in Ethiopia, India, Peru, and Vietnam.

**Method:**

Longitudinal data of 6601 children aged 1 to 15 years were obtained from the Young Lives cohort study. The data were analyzed using a latent basis growth curve model.

**Results:**

The findings of the study revealed that the rates of growth did not remain constant across the time intervals, which indicates the nonlinearity of the growth trajectory over time. For instance, children had the highest rate of growth between age 1 and 5 years, then between age 8 and 12 years, and a low rate of growth was observed between age 12 and 15 years. At the first measurement occasion (age 1 year) females were 0.826 cm (*p* < 0.0001) times shorter than males. The mean height at one year of age ranged from 72.13 cm in Ethiopia to 72.62 cm in India. Children in India and Vietnam had higher mean height at age one year. However, no significant difference in mean height at age one year was found between Ethiopian and Peruvian children, ($p=0.914$). Peruvian and Vietnamese children grew at a faster rate, while Indian children grew at a slower rate than Ethiopian children.

**Conclusion:**

We found substantial latent growth variations among children in four low- and middle-income countries. The latent trajectories differed by gender and country. The outcomes of the study could aid in detecting inequalities in children's height growth.

## Background

Child growth which is defined by a gain in weight, height and other measurements is a basic characteristics of development [1]. It is one of the most accurate indicators of population health, wellbeing, and living conditions and nutritional status in childhood [2, 3]. Furthermore, the assessment of children's growth patterns is important to understand the child anthropometric indices of growth. Children's stunting and underweight are associated with a lack of growth [4]. The socioeconomic status in which children grow up has a significant impact on their health and development [1]. For instance, children from low-income families have poorer health than children from higher-income families [5]. Due to the diverse ethnicity and cultural background of families, anthropometric measurements (height and weight) of healthy children differ in different areas of the world [6]. As a result, investigating variations in body height growth over time and across countries might help to detect inequalities in childhood living conditions [7].

When sufficient allowances are made for variations in genetic ability, average values of children's height and weight accurately represent the condition of a nation's public health and the average nutritional status of its people particularly in developing countries [8]. This study provides a detailed overview of differences in growth change among children measured height between 2002 and 2016 in four low- and middle-income countries. In the comparative study of children's physical growth in distinct populations, we are more interested in the average and variance of groups of children than in the growth trajectories of individuals. As a result, a well-designed growth model is an effective tool for examining differences in childhood growth patterns over time. Longitudinal studies are appropriate tools for a clear understanding of growth trajectories when each individual is measured repeatedly on the same outcome over many years [9, 10].

The growth trajectories are expected to demonstrate nonlinear change if followed for a long enough period span, as the outcome variable has a nonlinear association to time [11, 12]. In modeling such trajectories, defining the functional form of the mean pattern over time, which is commonly done by comparing models that tolerate nonlinearity with respect to time, as well as determining the amount to which individual growth trajectories differ around that mean pattern are crucial first tasks. To complete these tasks, growth curve models can be employed. Several authors have modeled the human growth curve in terms of mathematical functions. A few authors, for example, Karlberg [13] has proposed ICP-model (infancy, childhood and puberty) which split postnatal growth into three separate components which reflect the different biological/endocrinological periods, the Jenss-Bayley model [14], and the Reed model [15]. The growth process is a latent which is not observed directly. However, the main challenge of these models is that they cannot account for latent variables. To overcome this challenge, structural equation modeling [16] is used to fit growth curves in this study.

A structural equation modeling allows the repeated measurements (observed measurements) as multiple indicators on unobserved or latent factors to model unobserved growth trajectories [17]. Therefore, this study focuses on latent growth curve models in the context of a structural equation modeling framework to examine the gender and country contributions to variation in height growth from 1 to 15 years of age, using data from four low- and middle-income countries. The hypotheses of the study are: 1) The patterns of growth differ by gender, and 2) The patterns of growth differ by country.

## Methods

### Data source

Data from the Young Lives study were used for this study. The Young Lives research is a 15 years longitudinal cohort study that looked at how childhood poverty changed over time in Ethiopia, India, Peru, and Vietnam. The study has two cohorts of children. The older cohort of 1000 children born before the millennium development goals and the younger cohort of 2000 children born just after the millennium development goals were recruited from each country. The repeated measures of quantitative anthropometric data (height and weight) were gathered from older and younger cohorts, respectively, ranging in age from 8 to 22 years and 1 to 15 years. Five rounds of qualitative and quantitative data collecting were completed. The first round of the survey was performed in 2002 when children were on average 1 (younger cohort) and 8 (older cohort) years old; the second round was performed in 2006, the third in 2009, the fourth in 2013, and the fifth in 2016 [18]. Details regarding sampling and participant recruitment have been discussed in previously published work [19, 20]. Children in the younger cohort who had completed five rounds were included in this study.

### Latent basis growth curve model

The goal of a longitudinal study is to analyze phenomena in terms of their time-related constancy and change [21]. Thus, it is important to include time into a statistical model to obtain a better understanding of growth change over time. Statistical techniques that incorporate time as a factor or function in the model can be classified as a growth curve model [22]. Growth curve analysis represents the procedures of defining and establishing scientific inferences regarding the growth change and patterns of change in a wide range of time-related events [23]. This model openly displays outcomes as a function of time, making it ideal for examining systematic change in longitudinal data [24]. The latent growth curve model, as a subset of the broader structural equation modeling, can benefit from structural equation modeling's advantages. As such, there are numerous ways to extend the latent growth curve model: structured and unstructured latent growth curve model [24, 25]. The structured latent growth curve model has the advantage of being able to specify and test known functional forms of change across time, for instance, polynomial latent growth curve models. In contrast, latent basis or a shape-factor or freed-loading models determine the functional form for the change across time by estimating some basis function on the growth factors [21, 26].

A latent growth curve model assumes that underneath the observed outcomes for each subject is a latent trajectory, meaning that a true curve is not directly observable [16]. In such cases, the latent trajectory is described using a weighted combination of basis functions which reflects a shape of change that the underlying outcome is supposed to follow [25]. In order to account for the nonlinear patterns contained in the data, polynomial functions are predefined and included in a structured latent growth model. However, the unstructured latent technique describes nonlinearity in growth curves by estimating the basis function coefficients for the growth parameters [21]. In an unstructured latent model, the values of the basis functions are estimated from the data not predetermined. Hence, unstructured latent growth curve models gained the name called latent basis growth curve models [23]. A latent basis growth curve model is very flexible and can handle nonlinear trajectories by relaxing the basis function coefficients for the latent slope. The shape of the trajectory is latent in the sense that it is derived from data. That is, the form of the trajectory does not have to follow a predetermined functional form; instead, it is an optimal functional form derived from the data [27].

Growth change is examined as a function of time in a latent growth model, and it is characterized by the description of latent variables known as growth factors. Growth factors, therefore, provide a prediction of the mean trajectory, as well as individual differences around that trajectory [28]. For example, assume ${y}_{ti}$ is a set of responses for subject i at time point t, then the latent growth curve model with an estimated basis function can be expressed as:

1 $$\left.\begin{array}{c}{y}_{ti}={\alpha }_{i}+{\beta }_{i}{\Lambda }_{\mathrm{t}}+{\varepsilon }_{ti}\\ {\alpha }_{i}={\mu }_{\alpha }+{e}_{\alpha } \\ {\beta }_{i}={\mu }_{\beta }+{e}_{\beta }\end{array}\right\}$$

where ${\alpha }_{i}$ and ${\beta }_{i}$ are latent variables represent the initial and growth rate factor for i-th individual, respectively, ${\Lambda }_{\mathrm{t}}$ represents the basis function for the factor of growth rate, and ${\varepsilon }_{ti}$ is a time-specific residual. The latent variables ${\alpha }_{i}$ is stated as a function of a latent intercept ${\mu }_{\alpha }$ and ${\beta }_{i}$ is stated as a function of a latent slope ${\mu }_{\beta }$. The variance of the intercept factor ${e}_{\alpha }$ is used to model individual deviations from the group intercept, while the variance of the slope factor ${e}_{\beta }$ is used to represent individual deviations from the group slope [29]. Therefore, ${\alpha }_{i}$ and ${\beta }_{i}$ are random components that vary across subjects. The variance–covariance of the initial and growth rate are, respectively, ${\psi }_{\alpha \alpha }$, ${\psi }_{\beta \beta }$ and ${\psi }_{\alpha \beta }$.

In latent basis growth curve models, the unknown basis functions are computed from the data by specifying at least two basis function coefficients [21, 30]. The basis coefficients are the loadings from the latent slope to the repeated measures [31]. A common choice is to fix the first basis coefficient to zero (${\lambda }_{1}=0$) to establish an interpretation of the latent intercept as initial level and the last basis coefficient to ${\lambda }_{T}$ to allow interpretation of the pattern of loadings on the growth change [26]. For instance, for 5 waves of data, when the time scores are 0, 4, 7, 11, 14, the model would be:

2 $$\left[\begin{array}{c}{y}_{1i}\\ {y}_{2i}\\ {y}_{3i}\\ {y}_{4i}\\ {y}_{5i}\end{array}\right]=\left[\begin{array}{cc}1& 0\\ 1& {\lambda }_{2}\\ 1& {\lambda }_{3}\\ 1& {\lambda }_{4}\\ 1& 14\end{array}\right]\left[\begin{array}{c}{\alpha }_{i}\\ {\beta }_{i}\end{array}\right]+\left[\begin{array}{c}{\varepsilon }_{1i}\\ {\varepsilon }_{2i}\\ {\varepsilon }_{3i}\\ {\varepsilon }_{4i}\\ {\varepsilon }_{5i}\end{array}\right]$$

For identification purposes, the model specified 0 and 14 basis coefficients for the first and the last waves, respectively, while the other parameters are estimated freely from the data. The parameters ${\lambda }_{1},\dots ,{\lambda }_{T-1}$ are basis coefficients that determine the functional form of the trajectories. If the basis coefficients are equally spaced, it shows that the change in growth is linear, if not the change in growth is nonlinear [30, 31].

**Fig. 1:**
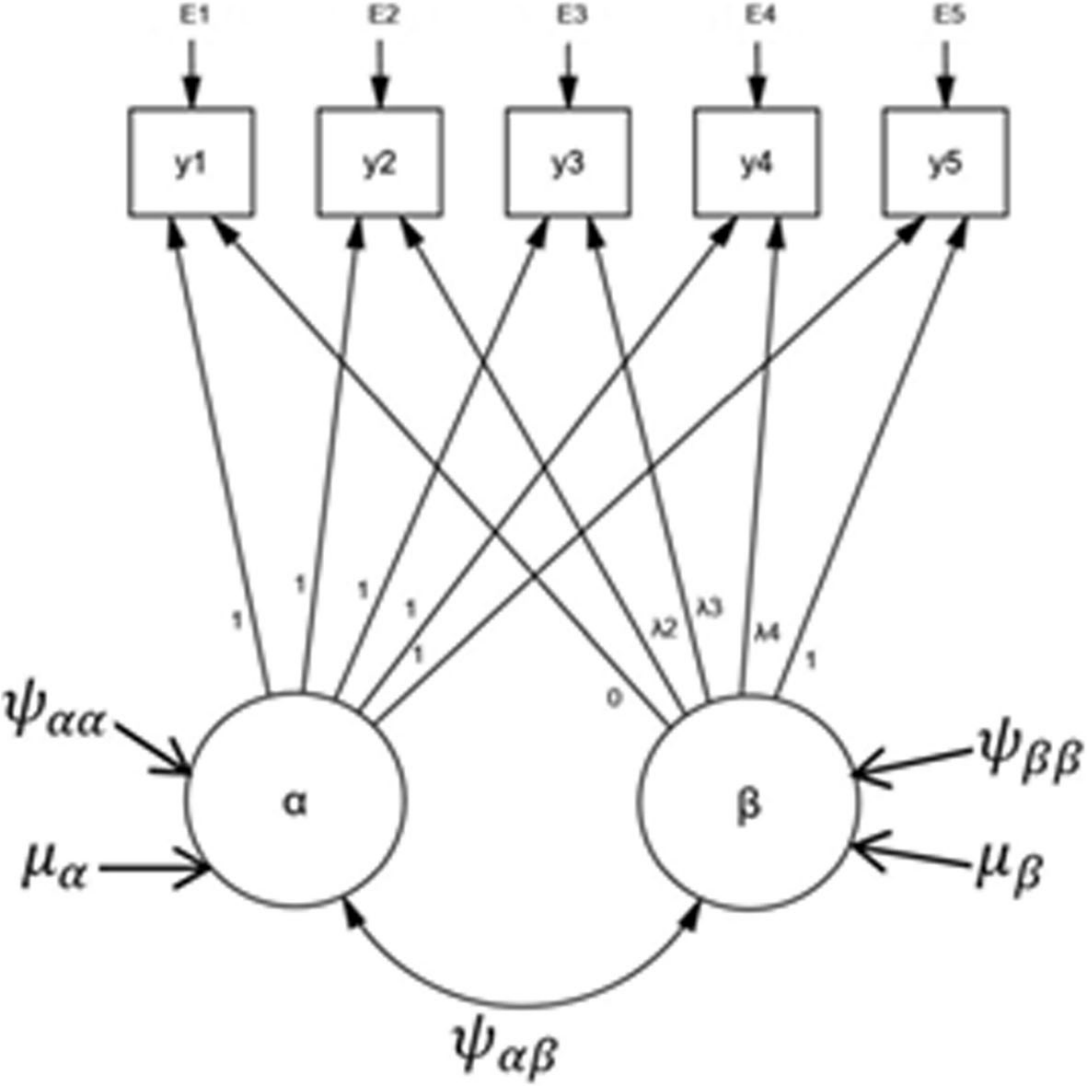
Path diagram for latent basis growth model with five waves of data

Figure 1 depicts a latent basis growth curve path diagram with 5 measurements of height data. The height measurements, ${(y}_{1},{y}_{2},\dots ,{y}_{5})$, are expressed as a function of the 2 latent components, $\alpha$ and $\beta$, and residuals, $\left({\varepsilon }_{1},{\varepsilon }_{2}\dots ,{\varepsilon }_{5}\right)$. The basis coefficients of $\alpha$ and $\beta$ are, respectively, fixed to 1 and $\left(0,{\lambda }_{2},{\lambda }_{3},{\lambda }_{4},1\right)$. The first time point ${t}_{1}$ in this specification corresponds to the initial of the height measurement. The interpretation of the initial measurement is determined by the origin of time points [32]. The origin, on the other hand, can be located at any point in time based on the objectives of the study. The latent components, the intercept ($\alpha$) and slope or shape factor $\left(\beta \right)$, have means (${\mu }_{\alpha }$ and ${\mu }_{\beta }$), variances (${\psi }_{\alpha \alpha }$ and ${\psi }_{\beta \beta }$), and a covariance (${\psi }_{\alpha \beta }$).

An intercept component describes the predicted value at time point zero. It is constant through time points, which is obtained by fixing basis functions of all time points on the latent intercept to 1 [29]. Identification basis coefficients must be placed on ${\Lambda }_{\mathrm{t}}$ to define the slope factor $\beta$. For instance, in Eq. (2), ${\Lambda }_{1}=0$ and ${\Lambda }_{\mathrm{T}}=14$, making intercept interpreted as individual’s predicted score at $t=1$ and shape factor $\beta$ as the total amount of change that occurred from $t=1$ to $t=T$. On the other hand, estimated basis coefficients from ${\Lambda }_{2}$ to ${\Lambda }_{5}$ denote the percentage of overall change that has occurred up to that point in time [33]. In addition to this, they showed departures of the trajectories from linearity [26]. To illustrate, consider that the basis function at time t is the sum of ${\lambda }_{t}$ and the amount by which the basis function deviates from linearity at time t, represented ${\theta }_{t}$ (i.e., ${\lambda }_{t}+{\theta }_{t}$). The following re-expression is given for the previous Eq. (2):

3 $$\left[\begin{array}{c}{y}_{1i}\\ {y}_{2i}\\ {y}_{3i}\\ {y}_{4i}\\ {y}_{5i}\end{array}\right]=\left[\begin{array}{cc}1& 0 \\ 1& 4 +{\theta }_{2}\\ 1& 7 +{\theta }_{3}\\ 1& 11+{\theta }_{4}\\ 1& 14 \end{array}\right]\left[\begin{array}{c}{\alpha }_{i}\\ {\beta }_{i}\end{array}\right]+\left[\begin{array}{c}{\varepsilon }_{1i}\\ {\varepsilon }_{2i}\\ {\varepsilon }_{3i}\\ {\varepsilon }_{4i}\\ {\varepsilon }_{5i}\end{array}\right]$$

Since ${\lambda }_{1}$ and ${\lambda }_{5}$ are pre-specified, ${\theta }_{1}={\theta }_{5}=0$. The rest, ${\theta }_{2},{\theta }_{3}$ and ${\theta }_{4}$ are estimated and used to solve for basis coefficients, ${\lambda }_{2},{\lambda }_{3}$ and ${\lambda }_{4}$, respectively. Equation (3) tells us about deviations of trajectories from linearity at time t. Generally, due to the lack of a specific functional form, a latent basis growth curve model can capture a wide range of nonlinear patterns [33].

Model fit was evaluated using comparative fit index (CFI), the Tucker-Lewis index (TLI), the root mean square error of approximation (RMSEA), the standardized root mean square (SRMS). The higher values closed to 1 for CFI and TLI reflecting a better fit, while the lower values closed to zero for RMSEA and SRMS reflecting a better fit [34, 35].

## Results

This study provides a detailed summary of differences in height growth among children in four low- and middle-income countries. Child height was measured at five different uneven time intervals of 1, 5, 8, 12, and 15 years and used as an observed variable in modeling a latent trajectory. All of the participants in this study were, on average, 1 year old at the time of the first measurement. Table 1 shows the means, standard deviations, and correlation matrix for the five measurement occasions of height data.

**Table 1:** Means and correlation matrix of height at five different measurement occasions

Height	Age 1	Age 5	Age 8	Age 12	Age 15
**Correlation matrix**					
Age 1	1.000				
Age 5	0.56711	1.000			
Age 8	0.50055	0.76121	1.000		
Age 12	0.41197	0.6636	0.75258	1.000	
Age 15	0.39373	0.55641	0.62422	0.57612	1.000
**Mean and standard deviation (SD)**					
Mean	71.9456	104.8436	120.7948	142.9084	157.4089
SD	4.7347	5.1106	5.6990	7.2132	7.2576

### Unconditional latent basis growth curve models

Unconditional latent basis growth curve models with different basis functions were employed. The coefficients in the basis function for the growth rate component,${\Lambda }_{\mathrm{t}}$, of Eq. (1) are fixed to specific values to indicate linear growth of the height data; for example, values matching to the measurement occasion ${\Lambda }_{\mathrm{t}}=\left(0, 4, 7, 11, 14\right)$ or any comparable scaling. However, under a latent basis growth curve model, the functional form of the trajectory is unknown and determined by estimating the values of the basis coefficients from the data. Therefore, models with varied basis functions were fitted to the height data: Model l: ${\Lambda }_{\mathrm{t}}=\left(0, 4,{\lambda }_{3}, {\lambda }_{4},{\lambda }_{5}\right)$ and Model 2: ${\Lambda }_{\mathrm{t}}=\left(0,{\lambda }_{2},{\lambda }_{3}, {\lambda }_{4},14\right)$. Fixing the basis coefficient of the first measurement to zero formally indicates that the initial status of height measurement at the age of one. Based on the objectives of the study, the initial status can be shifted to any time point [32]. Model 1 and 2 provide a different interpretation for the growth change parameter. In model 1, the latent intercept indicates the mean height at the first measurement. The latent slope, on the other hand, represents the growth change between the 1^st^ and the 2^nd^ measurements. In Model 2, the latent intercept still has the same interpretation as in model 1 since the origin of the time point is invariant in both models. However, the growth change is interpreted in terms of the full time.

Table 2 displays the models’ parameter estimates and fit statistics. The fitted growth models are statistically comparable and provide a satisfactory fit to the data (TLI = 0.998, CFI = 0.999, RMSEA = 0.025). Since the zero time point did not change in both models, the latent intercept's mean and variance are the same for both models (${\alpha =71.945, \psi }_{\beta \beta }=13.374$). The variations between the models are in the estimations of the slope, variance of slope and covariance. There must be discrepancies in the parameter estimates with varying basis function coefficients [24]. The purpose of these models is to clarify by demonstrating the challenge of scaling basis functions and to demonstrate the consequences for the estimated parameters and models fit. To elucidate this, the freely estimated basis coefficients in Model 1 are ${\Lambda }_{\mathrm{t}}=\left(0, 4, {\lambda }_{3}=5.94, {\lambda }_{4}=8.628, {\lambda }_{5}=10.391\right)$ and in Model 2 are ${\Lambda }_{\mathrm{t}}=\left(0, 5.389, 8.003, 11.624, 14\right)$. The freely estimated basis coefficients for the shape factor in Model 1 suggest that the change between age 5 and 8 years was 1.94, (i.e., 5.94—4), ​times the growth rate factor or overall change between infancy and middle adolescence. Similarly, the change between age 8 and 12 years was 2.688, $(i.e., 8.628-5.94)$, times the growth rate, and the change between age 12 and 15 was 1.763, $(i.e., 10.391-8.628)$, times the growth rate.

**Table 2:** Results of fitted two unconditional latent basis model

	**Model**			
	**Model 1**		**Model 2**	
	**Estimate**	**SE**	**Estimate**	**SE**
**Basis coefficient**				
${\lambda }_{1}$	0		0	
${\lambda }_{2}$	4		5.389	0.008
${\lambda }_{3}$	5.940	0.007	8.003	0.009
${\lambda }_{4}$	8.628	0.012	11.624	0.012
${\lambda }_{5}$	10.391	0.016	14	
**Mean**				
$\alpha$	71.945	0.058	71.945	0.058
$\beta$	8.225	0.014	6.105	0.006
**Variance**				
${\psi }_{\alpha \alpha }$	13.374	0.406	13.374	0.406
${\psi }_{\beta \beta }$	0.182	0.008	0.1	0.005
**Covariance**				
${\psi }_{\alpha \beta }$	0.057	0.048	0.043	0.036
**Index of fit**				
TLI	0.998		0.998	
CFI	0.999		0.999	
RMSEA	0.025		0.025	
AIC	49.784		49.784	

**Fig. 2:**
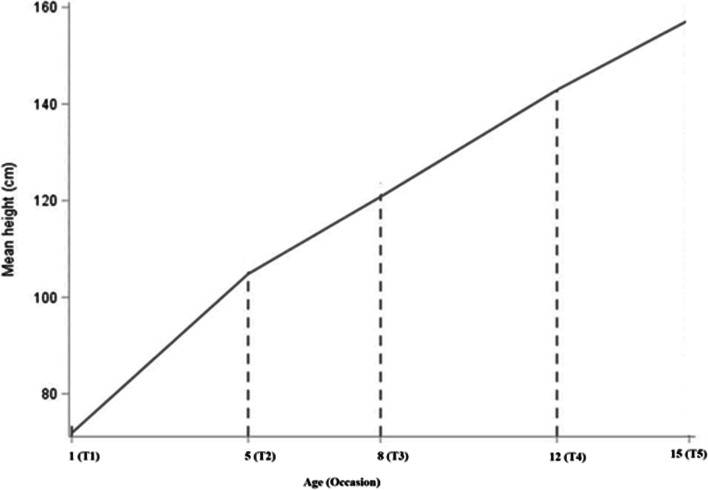
The mean growth patterns of children height aged 1 to 15

The study revealed that children had the highest change between age 1 and 5 years, next between age 8 and 12 years and get less growth between age 12 and 15 years. The results indicate that the increments of the basis coefficients are not constant reflects that the functional form of the growth trajectory is nonlinear. The estimated basis coefficients in Model 2, on the other hand, tell us the overall change between age 1 and 15 years as well as about the trajectory deviations from linearity. The mean rate of change from infancy to middle adolescence was 6.105. On the other hand, the results show that the deviation from linearity at the 2^nd^ occasion was about 1.389, (${i.e.,\theta }_{2}=5.389-4$), at the third occasion was about 1.003 ($i.e., {\theta }_{3}=8.003-7$) and at the fourth occasion was about 0.624 ($i.e.,{\theta }_{4}=11.624-11$). The values of all $\theta$’s are not equal, suggest that the functional form of height growth is not linear. Figure 2 further indicates the growth patterns of children height aged 1 to 15 years are not linear. The pattern line depicts a faster increase in growth from the first to the second measurement occasion and a slight increase in the subsequent time points. Therefore, the growth model with an unspecified functional form of latent growth is suitable to fit the data.

### Latent basis growth curve model with time-invariant covariates

There are different trends of change in height from infancy to adulthood. Therefore, individuals do not always react in the same fashion to growth and change over time. To resist the linear trend assumption, it is possible to freely estimate the slope basis function, as opposed to a latent growth curve model that specifies the slope basis function based on the measurement occasion used [33]. Therefore, to investigate the most likely growth change from age 1 to 15 years, Model 2 was employed with basis coefficients of the beginning and the end time points were specified (Fig. 2). Gender and country (country of children) were introduced as time-invariant covariates in the model. These covariates are assumed to affect the growth change and are therefore modeled as predictors of the growth factors. The results of this model reveal the initial level of height, the mean rate of change from infancy to middle adolescence (i.e., between age 1 and 15), and variance–covariance of the growth factors. Furthermore, the study discovered a difference in growth between females and males, as well as between four low- and middle-income countries. Table 3 shows the fit results for this model.

**Table 3:** Results of conditional latent basis model

	**Estimate**	**SE**	**CR**	***P*-value**
**Growth factor**				
$\alpha$	72.126	0.126	573.110	***
$\beta$	6.143	0.013	489.145	***
**Time-invariant covariate**				
$\alpha$<–- Gender (Female)	-0.826	0.111	-7.410	***
$\beta$<–- Gender (Female)	-0.090	0.011	-8.108	***
Country < –- Ethiopia (Reference group)				
$\alpha$<–- India	0.498	0.159	3.125	0.002
$\alpha$<–- Peru	0.017	0.160	0.108	0.914
$\alpha$<–- Vietnam	0.342	0.157	2.179	0.029
$\beta$<–- India	-0.143	0.016	-9.027	***
$\beta$<–- Peru	0.049	0.016	3.064	0.002
$\beta$<–- Vietnam	0.112	0.016	7.122	***
**Variance–covariance**				
${\psi }_{\alpha \alpha }$	12.935	0.386	33.538	***
${\psi }_{\beta \beta }$	0.073	0.004	17.357	***
${\psi }_{\alpha \beta }$	0.210	0.031	6.750	***

***Note:*** ***p < 0.0001

As seen in Table 3, the estimated initial component of 72.14 indicates the mean height of children at age one. Similarly, the estimated shape factor $\beta =6.143$ suggests that the rate of change in growth over time. Significant negative gender difference in height growth was observed at both growth factors. These suggest that females had significantly lower initial mean height and rate of growth ($\alpha =-0.83, \beta =-0.09$) than males. By incorporating countries as predictors of change in the growth model, the variations in the growth trajectories of children in four countries were analyzed. The study identified that county differences were significantly predicted the growth factors. The comparisons found that, at baseline, children in India and Vietnam showed positive and significant mean height ($\mathrm{India}:\alpha =0.498,p=0.002,$ $\mathrm{Vietnam}: \alpha =0.342,p=0.029$) when compared to Ethiopian children. This indicates that children in India and Vietnam were 0.418 and 0.342 times taller at age one than children in Ethiopia, respectively. However, no significant difference in mean height at the initial level was found between Ethiopian and Peruvian children, ($\alpha =0.017,p=0.914$). The positive and significant rate of change for children in Peru and Vietnam (Peru:$\beta =0.043,p=0.002$, Vietnam: $\beta =0.112,p<0.001$) suggests that children in Peru and Vietnam grew at a faster rate than children in Ethiopia. Children in India, on the other hand, exhibited a negative and significant rate of change ($\beta =-0.143,p<0.001$), indicates that Indian children grew at a slower rate than Ethiopian children.

Individual growth variability is represented by the variance–covariance of the model components. Both the latent intercept (${\psi }_{\alpha \alpha }=12.94,p<0.001$) and the latent slope (${\psi }_{\beta \beta }=0.07,p<0.001$) had significant variance, indicate that there were significant individual differences in initial height and rate of change. Finally, the rate of growth and the mean intercept of height was positively correlated (${\psi }_{\alpha \beta }=0.21,p<0.001$). This indicates that children who reported higher initial values of height tended to show a slight increase in growth over time compared with children who reported lower initial values of height.

## Discussion

Growth changes are complicated phenomena that require the use of robust models to depict and comprehend them. A prominent method for examining growth trajectories is to model change using structured and unstructured latent growth curve models within a structural equation modeling framework [36]. The structured latent growth model assumes that change happens in a specific pattern, for example, in either linear, quadratic, or cubic patterns. Nevertheless, accurately modeling the real process of change using a specific pattern may be problematic [37]. Due to these problems, we considered unstructured latent change analysis to model the current data since it does not enforce a particular functional form depending on the pattern of change appearing in the data.

The main goal of this study was to look at how the height of children in four low- and middle-income countries varies over time and across countries. To find the underlying functional form of growth patterns in the latent growth analysis, we first constructed the unconditional latent model excluding potential factors. Thus, the two unconditional latent basis models with different basis functions were fitted, and then the conditional latent basis model was fitted. In the first unconditional model, the first and the second basis coefficients were specified to represent the mean height at the first measurement occasion and the change of growth between the first and second measurement occasion, respectively. Our preliminary analysis revealed that a linear growth model did not adequately fit the data, implying that the growth curve contains nonlinear change. The highest growth change occurred between ages 1 and 5, while the lowest occurred between ages 12 and 15. This finding is consistent with previous studies [38–40].

Another essential aspect of this study was to investigate the relations of gender and country variables with child growth and then we included these time-invariant covariates as the predictors of the growth factors. The time-invariant effect of gender on the latent intercept represented that females reported shorter height on average compared to males. Similarly, there was a gender effect on shape factor, with males growing at a faster rate than females. Furthermore, results of the conditional latent basis model with the time-invariant effect of the country showed that India and Vietnam had a higher mean height at age one year than children in Ethiopia. However, there was no significant difference in mean height at age one year between Ethiopian and Peruvian children. In our previous study, we applied a piecewise mixed-effects approach to examine gender and country variations in the height growth at different growth phases. It was found that variations in height growth are significant [41]. Using the same datasets of the Young Lives cohort study, a previous study investigated child growth trajectories and found that children in Ethiopia had a lower mean height-for-age Z-score (HAZ) at age one year, whereas children in Vietnam had a higher HAZ at age one year [42].

We also analyzed the difference in the mean rate of change across four countries for 15-years trajectories. Children in four countries had substantial variations in growth change. Peruvian and Vietnamese children grew at a higher rate than Ethiopian children, whereas Indian children grew at a slower rate. This may be due to differences in socioeconomic level among countries. The previous study, which utilized the same datasets of the Young Lives cohort study, found that height disparities resulting from early-life situations remain even when children reach early adolescence [1]. According to a study conducted by Bassino [43], regional income inequality in Japan between 1892 and 1941 explains inequalities in population mean height. Children of higher socioeconomic status were taller than those of lower socioeconomic status. Children from higher socioeconomic status were taller than those from lower socioeconomic status [44].

## Conclusion

This study examined the latent change process in height growth of children aged 1 to 15 years in four low- and middle-income countries. Furthermore, the effects of gender and country differences on child growth were investigated. It was found that the functional form between child growth and age is nonlinear, with rapid growth change observed between age 1 and 5 years. The outcomes of the study may help to identify inequities in children's living conditions.

## Data Availability

The datasets analyzed during the current study are available in the Young Lives study repository, http://www.younglives.org.uk/.
